# Heterogeneity of the HIV epidemic in the general population of Karnataka state, south India

**DOI:** 10.1186/1471-2458-11-S6-S13

**Published:** 2011-12-29

**Authors:** Pradeep Banandur, Subramanian Potty Rajaram, Sangameshwar B Mahagaonkar, Janet Bradley, Banadakoppa M Ramesh, Reynold G Washington, James F Blanchard, Stephen Moses, Catherine M Lowndes, Michel Alary

**Affiliations:** 1 CHARME II Project, Bangalore 560044, India; 2 Department of Community Medicine, Rajarajeswari Medical College and Hospital, Bangalore 560074, India; 3 CHARME I Project, Bangalore 560044, India; 4 Karnataka Health Promotion Trust, Bangalore 560044, India; 5 Infectious Diseases Unit, St John’s Research Institute, Bangalore 560034, India; 6 University of Manitoba, Winnipeg, Canada; 7 Health Protection Agency, London, UK; 8 URESP, Centre de recherche du CHA universitaire de Québec, Québec, Canada; 9 Département de médecine sociale et préventive, Université Laval, Québec, Canada

## Abstract

**Background:**

In the context of *AVAHAN*, the India AIDS Initiative of the Bill & Melinda Gates Foundation, general population surveys (GPS) were carried out between 2006 and 2008 in Belgaum (northern), Bellary (mid-state) and Mysore (southern) districts of Karnataka state, south India. Data from these three surveys were analysed to understand heterogeneity in HIV risk.

**Methods:**

Outcome variables were the prevalence of HIV and sexually transmitted infections (STIs). Independent variables included age, district, place of residence, along with socio-demographic, medical and behavioural characteristics. Multivariate logistic regression was undertaken to identify characteristics associated with HIV and differences between districts, incorporating survey statistics to consider weights and cluster effects.

**Results:**

The participation rate was 79.0% for the interview and 72.5% for providing a blood or urine sample that was tested for HIV. Belgaum had the highest overall HIV (1.43%) and *Herpes simplex* type-2 (HSV-2) (16.93%) prevalence, and the lowest prevalence of curable STIs. In Belgaum, the HIV epidemic is predominantly rural, and among women. In Bellary, the epidemic is predominantly in urban areas and among men, and HIV prevalence was 1.18%. Mysore had the lowest prevalence of HIV (0.80%) and HSV-2 (10.89%) and the highest prevalence of curable STIs. Higher HIV prevalence among men was associated with increasing age (p<0.001), and with history of STIs (AOR=2.44,95%CI:1.15-5.17). Male circumcision was associated with lower HIV prevalence (AOR=0.33,95%CI:0.13-0.81). Higher HIV prevalence among women was associated with age (AOR_25-29years_=11.22,95%CI:1.42-88.74, AOR_30-34years_=13.13,95%CI:1.67-103.19 and AOR_35-39years_=11.33,95%CI:1.32-96.83), having more than one lifetime sexual partner (AOR=4.61,95%CI:1.26-16.91) and having ever used a condom (AOR=3.32,95%CI:1.38-7.99). Having a dissolved marriage (being widowed/divorced/separated) was the strongest predictor (AOR=10.98,95%CI: 5.35-22.57) of HIV among women. Being a muslim woman was associated with lower HIV prevalence (AOR=0.27,95%CI:0.08-0.87).

**Conclusion:**

The HIV epidemic in Karnataka shows considerable heterogeneity, and there appears to be an increasing gradient in HIV prevalence from south to north. The sex work structure in the northern districts may explain the higher prevalence of HIV in northern Karnataka. The higher prevalence of HIV and HSV-2 and lower prevalence of curable STIs in Belgaum suggests a later epidemic phase. Similarly, higher prevalence of curable STIs and lower HIV and HSV-2 prevalence in Mysore suggests an early phase epidemic.

## Introduction

HIV epidemics can be highly heterogeneous [1], and such heterogeneity is observed globally [2]. Both the African and Asian regions have shown vast heterogeneity in HIV prevalence between and within countries [3,4]. India contributes to almost 60% of South Asia’s HIV epidemic [4], with an estimated 2.4 million people living with HIV in India [5]. Within India, HIV is concentrated in four southern states, where heterosexual transmission is primarily responsible for transmission [4,5], as well as in some northeastern states [4,5], where the epidemic is driven largely by injection drug use [4]. In the southern states, HIV prevalence varies between and within districts, between sub-districts, and even between villages [6]. Karnataka is one such state, where northern Karnataka districts such as Belgaum and Bagalkot have consistently shown higher HIV prevalence among antenatal clinic (ANC) populations, while the southern district of Mysore has relatively low antenatal HIV prevalence [7]. The prevalence of HIV among ANC attenders in Bellary district in central Karnataka seems to be intermediate [7]. There seems to be an increasing gradient from south to north in the prevalence of HIV in Karnataka. In addition, there is heterogeneity observed within districts, with HIV prevalence higher in rural than in urban areas in Bagalkot, while the opposite has been observed in Mysore [6,8]. However, no comparative analysis regarding the true general population prevalence of HIV has been carried out. In the context of *AVAHAN*, the India AIDS Initiative of the Bill & Melinda Gates Foundation, general population surveys (GPS) have been carried out in three Karnataka districts. Given the north-south gradient already observed in HIV prevalence in the antenatal populations in Karnataka districts, we analysed data from the three GPS surveys to better understand the heterogeneity of HIV and its determinants. The three districts are Belgaum (northern Karnataka), Bellary (middle of the state) and Mysore (southern Karnataka).

## Methods

We conducted a comparative analysis of data from three general population surveys conducted between 2006 and 2008 in the districts of Mysore, Belgaum and Bellary. The survey methodology adopted was similar in all the three districts.

### Research population and sampling

The target sample in each district was 6000 randomly selected individuals, equally distributed between rural and urban areas, and between men and women aged 15-49 years. In each district, a complete household census was conducted in 15 randomly selected rural villages and 20 randomly selected urban blocks (primary sampling units or PSUs). This census served as the sampling frame for selecting the required number of respondents for the main survey. For each of the urban and rural PSUs, the sampling frame consisted of persons who were aged 15-49 years, who were usual residents of the household, and who had stayed within the household the night before the census. This list was stratified by sex, age and marital status, and the required number of individuals in each PSU was selected systematically with probability proportionate to size. The number of respondents varied according to the actual population size in rural PSUs. In urban PSUs, a fixed number of 150 respondents were selected, since the urban PSUs generally had similar population size. In case of urban PSUs having less than 150 persons, the census list was attached to another urban PSU with a larger population size, and 300 persons were selected from the combined list. In each of the districts we had only one such case. The time periods of data collection for the main surveys were March to November 2006 in Mysore, March to July 2007 in Belgaum, and November 2007 to April 2008 in Bellary.

### Procedures for data collection

Data were collected using a structured pretested questionnaire, developed separately for male and female respondents, by trained field investigators of the same sex, at a place where the respondent felt comfortable, ensuring adequate privacy and confidentiality. Separate teams were formed considering the geographical extent of the districts. Each team consisted of one field supervisor, two male field investigators, two female field investigators, and a laboratory technician. The number of teams varied from a minimum of 4 in Mysore to a maximum of 10 in Belgaum. Field investigators administered a questionnaire after written informed consent. Written informed consent was obtained from each participant separately for face-to-face interviews and for collection of biological samples. Blood (venipuncture) and urine samples were collected from all consenting participants. Dried blood spots (DBS) were collected from those who did not consent for venipuncture, but consented to provide DBS. Collected samples were transported in cold boxes to laboratories at Rotary-TTK and St. John’s Medical College, Bangalore.

Institutional ethical review boards of St John’s Medical College, Bangalore, India, University of Manitoba, Canada, and Centre hospitalier *affilié* universitaire de Québec, Canada, approved the study. In addition, regulatory approvals were obtained from the Health Ministry Screening Committee, Indian Council of Medical Research, Government of India.

### Laboratory methods

Serum samples were tested for HIV, syphilis and *Herpes simplex* type 2 (HSV-2) antibodies, and urine samples were tested for chlamydial infection and gonorrhea. HIV testing was done using two different ELISA test kits in series. For Belgaum and Mysore, the first ELISA test was done using J Mitra kits (J. Mitra and Company Private Limited, New Delhi, India). In Bellary, the first ELISA test was done using the Detect HIV kit, (Adaltis Inc, Canada). All samples positive for the first ELISA test were tested using Genedia kits (Greencross Lifescience Corp, Kyunggi-do, South Korea). DBS samples were also tested using a similar testing procedure after elution. Those samples which tested positive with both tests were considered HIV positive. Respondents who did not give a blood sample, but did provide urine, were tested for HIV-1 antibodies in urine using an ELISA test (Calypte Biomedical Corporation, Berkeley, California, USA) with confirmation of initially positive results by urine Western Blot, provided by the same company. Serum samples were tested for syphilis antibodies using a rapid plasma reagin (RPR) test (Span Diagnostics, Surat, India). All samples positive for RPR were then tested with a *Treponema pallidum* haemagglutination (TPHA) test (Glaxo-Omega, Alloa, Scotland, United Kingdom). A respondent was considered positive for active syphilis when both the RPR and TPHA tests were positive. HSV-2 testing was carried out on a 1:8 sub-sample of randomly selected serum samples using the ELISA test from Kalon Biological (Surrey, United Kingdom). Testing for *Neisseria gonorrhoeae* (NG) and *Chlamydia trachomatis* (CT) was done using nucleic acid amplification tests (Aptima Combo 2 Assay, Gen-Probe, San Diego, CA, USA). Testing for HIV, HSV-2 and syphilis were conducted at St. John’s Research Institute, St. John’s Medical College, Bangalore. Testing for chlamydial infection and gonorrhea was conducted at Rotary-TTK Blood Bank, Bangalore.

### Statistical analyses

Prevalence estimates for HIV and sexually transmitted infections (STIs) were weighted according to sampling and population distributions. Weights were separately calculated for biological specimens and for the questionnaire items. The weights represented the inverse of the probability of selection. The final weight used was adjusted for the effect of different types of non-responses in each primary sampling unit. This took into account the non-response for place of residence as well. The sample weights were normalized so that the total weighted sample size was equal to the unweighted one. We compared these prevalence estimates across districts, rural vs urban areas and gender.

Univariate analysis of risk factors for HIV infection was carried out using logistic regression, incorporating survey statistics to consider weights and cluster effects. The prevalences of HIV and STIs were the outcome variables, and independent variables included age, as well as social, demographic, medical and behavioural characteristics. We tested associations between HIV prevalence and: age, literacy, marital status, number of lifetime partners, age at first sexual intercourse, ever having paid for/received payment for sex, history of anal sex, blood transfusion, injecting drug use, history of any STI ever in the past and religion for both men and women. In addition, we tested for circumcision and history of sex with other men among men. Separate models were fit for men and women. Those variables with a p-value <0.10 in the univariate analyses were included in the multivariate models. Both models were adjusted for district and place of residence (rural vs urban). We also tested for interaction between districts and independent variables in the final model, to identify similarities and differences in risk factors for HIV across districts. All analyses were carried out using STATA IC 10.1 (Stata Corporation, Texas) software.

## Results

### Response rates

Overall, 14,215 (79.0%) of those selected for survey provided interview, 12,759 (70.9%) provided a urine sample and 12,180 (67.7%) provided a serum/DBS sample in the 3 districts combined. A total of 13,039 subjects (72.5%) provided at least one biological sample (blood or urine) that was tested for HIV. Belgaum had the highest participation rate (82.1% for the interview and 76.5% for biological sample) compared to Bellary (77.3% for the interview and 71.0% for biological sample) and Mysore (77.6% for the interview and 69.9% for biological sample). We compared participants with non-participants in the survey for socio-demographic variables collected when carrying out the census, and found no difference according to age and marital status. There was however significant differences in participation rates by sex (men: 73.7% vs women: 84.4%, p-value<0.0001) and place of residence (urban: 75.0% vs rural: 83.0%, p-value<0.0001). Similar differences were observed when those who provided biological samples were compared with those who did not provide any biological sample.

### Variations between HIV and STI prevalence across districts, place of residence and gender

Table 1 shows that Belgaum district had the highest overall HIV prevalence. Belgaum also had the highest rural HIV prevalence and the lowest urban HIV prevalence. The highest urban HIV prevalence was seen in Bellary. HIV prevalence was higher among men than women in Bellary and Mysore, while in Belgaum, it was higher in women compared to men. Rural women had a higher HIV prevalence than rural men in Belgaum and Bellary, although this was not statistically significant. Urban HIV prevalence was higher among men compared to women in all three districts. HIV was more prevalent in the age group 25-39 years in all the three districts (Table 2). In Belgaum, rural HIV prevalence was significantly higher than urban HIV prevalence overall and in all age groups. In Mysore, urban prevalence was higher in the population aged below 35 years, while rural prevalence was higher among older subjects. In Bellary, rural and urban prevalences were similar among people aged less than 30 years, but prevalence in urban areas was higher in subjects aged 30 and above.

**Table 1:** Gender specific prevalence of HIV by district and place of residence^*^

	N	Belgaum (N=4578) % (95% CI)	Bellary (N=4258) % (95% CI)	Mysore (N=4190) % (95% CI)	P-value^$^
**All total**	13026	1.43 (0.86-2.01)	1.18 (0.74-1.62)	0.80 (0.50-1.09)	0.039
**Males**	6550	1.28 (0.51-2.06)	1.24 (0.70-1.78)	0.98 (0.45-1.52)	0.378
**Females**	6476	1.58 (0.94-2.23)	1.13 (0.59-1.66)	0.65 (0.28-1.02)	0.066
**Urban Total**	6060	0.63 (0.18-1.09)	1.36 (0.65-2.07)	0.94 (0.49-1.39)	0.049
**Urban Males**	2892	0.69 (0.25-1.12)	1.64 (0.71-2.56)	1.06 (0.35-1.78)	0.139
**Urban Females**	3168	0.58 (0.00-1.16)	1.12 (0.37-1.86)	0.84 (0.34-1.35)	0.310
**Rural Total**	6966	1.69 (0.90-2.49)	1.05 (0.46-1.64)	0.71 (0.31-1.10)	<0.0001
**Rural Males**	3318	1.48 (0.41-2.55)	0.97 (0.29-1.65)	0.93 (0.13-1.74)	0.030
**Rural Females**	3648	1.90 (1.01-2.79)	1.13 (0.34-1.93)	0.51 (0.00-1.05)	0.002

*All figures in weighted percentages, 95% CI: 95% Confidence interval

^$^ p-value based on Pearson’s χ^2^ test for difference across districts with two degrees of freedom

**Table 2:** Age-specific HIV prevalence by district and place of residence

	Age	15-19	20-24	25-29	30-34	35-39	40-44	45-49	Total
Belgaum p<0.0001	%	0.07	0.51	1.6	3.16	3.21	0.74	0.9	1.43
	CI	0.0-0.20	0.0-1.30	0.64-2.57	1.16-5.16	0.62-5.79	0.0-1.49	0.11-1.70	0.86-2.01
	N	831	796	717	611	694	459	470	4578

Belgaum Rural P<0.0001	%	0.09	0.7	1.62	3.68	3.96	0.85	1.02	1.69
	CI	0.0-0.28	0.0-1.85	0.39-2.85	1.0-6.35	0.40-7.51	0.0-1.85	0.0-2.08	0.9-2.49
	N	441	390	379	330	377	257	260	2434

Belgaum Urban p=0.094	%	0	0	1.56	1.47	0.77	0.38	0.51	0.63
	CI	NE	NE	0.0-3.28	0.0-3.91	0.0-1.68	0.0-1.18	0.0-1.52	0.18-1.09
	N	390	406	338	281	317	202	210	2144

Bellary p=0.004	%	0.13	1.08	0.97	2.43	2.36	0.41	1.48	1.18
	CI	0.0-0.31	0.29-1.86	0.13-1.80	0.48-4.38	0.94-3.78	0.0-1.01	0.10-2.86	0.74-1.62
	N	816	813	736	548	547	405	393	4258

Bellary Rural p=0.031	%	0.09	1.08	1	2.07	2.4	0	1.06	1.05
	CI	0.0-0.28	0.0-2.29	0.0-2.40	0.0-5.32	0.21-4.58	NE	0.0-2.73	0.46-1.64
	N	462	437	377	275	302	209	207	2269

Bellary Urban p=0.182	%	0.19	1.07	0.91	2.89	2.3	0.94	2.07	1.36
	CI	0.0-0.58	0.05-2.09	0.0-1.84	0.75-5.02	0.51-4.09	0.0-2.40	0.0-4.57	0.65-2.07
	N	354	376	359	273	245	196	186	1989

Mysore p=0.841	%	0.44	0.61	1.45	0.57	1.22	1.11	0.15	0.8
	CI	0.0-0.97	0.0-1.41	0.24-2.66	0.0-1.32	0.03-2.41	0.0-2.66	0.0-0.37	0.52-1.09
	N	693	771	700	580	598	433	415	4190

Mysore Rural p=0.912	%	0.34	0.3	1.42	0.1	1.38	1.23	0.23	0.71
	CI	0.0-1.06	0.0-0.77	0.0-3.32	0.0-0.31	0.0-3.20	0.0-3.87	0.0-0.57	0.32-1.10
	N	366	392	387	297	340	235	246	2263

Mysore Urban p=0.791	%	0.58	1.01	1.51	1.18	0.97	0.94	0	0.94
	CI	0.0-1.47	0.0-2.74	0.22-2.81	0.0-2.84	0.0-2.36	0.0-2.33	NE	0.49-1.39
	N	327	379	313	283	258	198	169	1927

Overall p<0.0001	%	0.19	0.67	1.4	2.15	2.39	0.78	0.76	1.17
	CI	0.02-0.36	0.22-1.13	0.81-1.99	1.13-3.18	1.11-3.68	0.19-1.38	0.31-1.22	0.88-1.47
	N	2340	2380	2153	1739	1839	1297	1278	13026

Overall rural p<0.0001	%	0.16	0.66	1.43	2.38	2.92	0.79	0.77	1.28
	CI	0.0-0.36	0.07-1.25	0.61-2.25	0.90-3.86	1.06-4.79	0.0-1.63	0.20-1.34	0.86-1.70
	N	1269	1219	1143	902	1019	701	713	6966

Overall urban p=0.044	%	0.96	0.27	1.34	1.72	1.24	0.77	0.75	0.96
	CI	0.0-0.60	0.0-1.43	0.60-2.08	0.61-2.84	0.50-1.99	0.07-1.46	0.0-1.53	0.66-1.26
	N	1071	1161	1010	837	820	596	565	6060

CI: 95% Confidence interval, All percentages and confidence intervals are weighted, NE: Not estimated

p-values for Pearson χ^2^ test for difference across age groups with six degrees of freedom

Overall, the prevalence of bacterial STIs was highest in Mysore. The prevalences of both chlamydia and syphilis were higher in Mysore than in the two other districts (chlamydia 1.05% in Mysore vs. 0.24% for Bellary/Belgaum, p<0.0001; and syphilis 1.36% in Mysore vs. 0.81% in Bellary/Belgaum, p=0.063). There were only six cases of gonorrhea overall (five in Mysore, one in Bellary). HSV-2 prevalence was higher in Belgaum overall and in rural areas compared to Bellary and Mysore (Table 3), but urban HSV-2 prevalence was lowest in Belgaum. However, these differences in HSV-2 prevalence were not statistically significant. The prevalence of HSV-2 antibodies was higher in women than in men in all three districts and these differences were statistically significant in Belgaum and Bellary (p=0.01).

**Table 3:** Gender specific prevalence of HSV-2 infection by district and place of residence^*^

	N	Belgaum (N=502) % (95% CI)	Bellary (N=475) % (95% CI)	Mysore (N=436) % (95% CI)	P-value^$^
Urban Total	652	7.54 (3.91-11.17)	10.10 (7.15-13.05)	9.56 (4.09-15.03)	0.406
Urban Males	322	4.00 (0.55-7.45)	6.37 (1.49-11.24)	7.98 (1.97-13.98)	0.318
Urban Females	330	11.53 (5.67-17.39)	13.33 (6.82-19.85)	11.01 (4.12-17.89)	0.835
Rural Total	761	19.87 (13.87-25.87)	12.31 (8.66-15.96)	11.80 (5.58-18.03)	0.452
Rural Males	367	11.43 (4.18-18.69)	7.37 (2.27-12.47)	9.64 (2.66-16.63)	0.834
Rural Females	394	27.96 (17.10-38.83)	17.09 (11.38-22.80)	13.83 (3.24-24.41)	0.431
All total	1413	16.93 (12.25-21.61)	11.38 (8.93-13.82)	10.89 (6.54-15.23)	0.929
Males	689	9.55 (4.09-15.01)	6.96 (3.51-10.41)	8.97 (4.56-14.09)	0.601
Females	724	24.29 (16.37-32.20)	15.46 (11.25-19.66)	12.67 (6.12-19.21)	0.707

*All figures in weighted percentages, 95%CI: 95% Confidence interval

^$^p-values for Pearson χ^2^ test for difference across districts with two degrees of freedom

### Analysis of risk factors for HIV

In multivariate analyses, increasing age, circumcision and ever having had an STI were significantly associated with HIV among men whereas having ever paid for sex was borderline significant (Table 4). For women, significant predictors of HIV positivity included age 25-40, having a dissolved marriage (i.e. widowed, separated or divorced), being of Muslim religion, having had more than one sexual partner during lifetime, and having ever used a condom (Table 5). Because of the relatively low HIV prevalence observed among older women, there was no overall trend related to age and HIV prevalence as observed among men. Among the risk factors identified, being widowed, divorced or separated was the strongest predictor of HIV positivity, with an adjusted odds ratio of around 11. In both men and women there was no significant interaction between districts and the other variables included in the model (all p-values >0.15).

**Table 4:** Multivariate analysis of factors associated with HIV positivity among men in three districts in Karnataka, 2005-2007

Characteristic	HIV prevalence % (95% CI)	Crude OR (95% CI) N=6210	Adjusted OR^*^ (95% CI) N=6210	P-value
**Age^$^**				
15-19 years	0.29 (0.0-0.59)	1.00 (Ref)	1.00 (Ref)	
20-24 years	0.60 (0.20-1.00)	2.05 (0.59-7.08)	2.02 (0.59-6.98)	0.260
25-29 years	1.13 (0.17-2.09)	3.87 (1.04-14.38)	3.66 (0.99-13.48)	0.051
30-34 years	1.58 (0.26-2.90)	5.45 (1.45-20.40)	5.24 (1.44-19.14)	0.013
35-39 years	2.80 (0.38-5.23)	9.78 (2.75-34.77)	9.45 (2.59-34.39)	0.001
40-44 years	1.45 (0.21-2.69)	5.00 (1.31-19.04)	4.57 (1.18-17.61)	0.028
45-49 years	1.19 (0.35-2.03)	4.07 (1.20-13.77)	3.87 (1.13-13.30)	0.032
**Undergone circumcision**				
**No**	1.27 (0.82-1.72)	1.00 (Ref)	1.00 (Ref)	
**Yes**	0.46 (0.07-0.86)	0.36 (0.14-0.92)	0.33 (0.13-0.81)	0.016
**Ever paid for sex**				
**No**	1.11 (0.72-1.51)	1.00 (Ref)	1.00 (Ref)	
**Yes**	4.57 (0.24-8.90)	4.26 (1.51-12.03)	2.68 (0.98-7.34)	0.055
**Ever had any STI**				
**No**	1.11 (0.72-1.51)	1.00 (Ref)	1.00 (Ref)	
**Yes**	3.13 (0.37-5.89)	2.86 (1.10-7.42)	2.44 (1.15-5.17)	0.021

%: Weighted percentages, 95% CI: 95% Confidence interval, OR: Odds ratio, Ref: Reference group

^*^Each variable is adjusted for district, place of residence (rural vs urban) and all the other variables shown in the table

^$^Test for trend significant at p<0.0001 level

**Table 5:** Multivariate analysis of factors associated with HIV positivity among women in three districts in Karnataka, 2005-2007

Characteristic	Prevalence % (95% CI)	Crude OR (95% CI) N=6816	Adjusted OR* (95% CI) N=6812	P-value
**Age**				
15-19 years	0.09 (0.0-0.26)	1.00 (Ref)	1.00 (Ref)	
20-24 years	0.76 (0.0-1.52)	8.71 (0.94-81.14)	5.85 (0.64-53.93)	0.118
25-29 years	1.63 (0.80-2.46)	18.85 (2.38-149.19)	11.22 (1.42-88.74)	0.022
30-34 years	2.63 (1.08-4.18)	30.75 (3.98-237.46)	13.13 (1.67-103.19)	0.015
35-39 years	2.07 (0.73-3.40)	24.03 (2.97-194.27)	11.33 (1.32-96.83)	0.027
40-44 years	0.19 (0.0-0.56)	2.16 (0.13-35.71)	0.82 (0.05-13.53)	0.889
45-49 years	0.34 (0.0-0.77)	3.88 (0.36-41.40)	1.44 (0.13-16.12)	0.767
**Marital Status**				
Others	0.67 (0.38-0.96)	1.00 (Ref)	1.00 (Ref)	
Marriage dissolved	7.20 (4.33-10.80)	11.58 (6.02-22.26)	10.98 (5.35-22.57)	<0.0001
**Number of sexual partners**				
One	1.08 (0.75-1.40)	1.00 (Ref)	1.00 (Ref)	
More than one	9.72 (0.83-18.62)	10.19 (3.66-28.42)	4.61 (1.26-16.91)	0.022
**Condom use**				
Never used condom	1.06 (0.74-1.38)	1.00 (Ref)	1.00 (Ref)	
Ever used condom	2.81 (0.49-5.12)	2.70 (1.11-6.54)	3.32 (1.38-7.99)	0.008
**Religion**				
Non-Muslim	1.27 (0.88-1.65)	1.00 (Ref)	1.00 (Ref)	
Muslim	0.28 (0.0-0.60)	0.22 (0.07-0.73)	0.27 (0.08-0.87)	0.029

%: Weighted percentages, 95%CI: 95% Confidence Interval, OR: Odds Ratio, Ref: Reference group

^*^Each variable is adjusted for district, place of residence (rural vs urban) and all the other variables shown in the table

Circumcision among men (Table 4), and being a Muslim among women (Table 5) were significantly protective for HIV. Circumcision was very highly correlated with religion (ρ=0.9515). In the model excluding circumcision but including religion, being a Muslim was protective among men (AOR=0.35, 95%CI: 0.15-0.86). We evaluated the association between religion and HIV risk behaviors (Eg: number of sexual partners in life time, ever had paid/received money for sex, ever had anal sex and condom use during last sex) among both men and women. For men, the only significant difference was that Muslim men reported more often than others having ever paid for sex (3.05% vs 1.83%, p<0.0001). There was no significant difference in HIV risk behavior between Muslim and non-Muslim women.

## Discussion

The HIV epidemic and patterns of other STIs in these three districts of Karnataka show considerable heterogeneity. To our knowledge, this is the first comparative analysis carried out using population- based survey data to understand the heterogeneity of the HIV epidemic within a state in India.

The observed south-north gradient for HIV observed among the prenatal population [7] is confirmed by this analysis. The district of Belgaum in northern Karnataka had the highest overall HIV prevalence among the three districts studied. The HIV epidemic in Belgaum is characterized by being more rural than urban, and is more advanced among women. The prevalence of HSV-2 was also highest in Belgaum. A high HIV prevalence in rural areas was reported in a similar general population survey conducted in the neighbouring district of Bagalkot [6]. The southern Karnataka district of Mysore had a much lower prevalence of HIV and HSV-2, but had the highest prevalence of bacterial STIs of the three districts. Rural and urban prevalences were almost similar for STIs and HIV in Mysore. Bellary (in central Karnataka) had a HIV prevalence which is intermediate between Belgaum and Mysore, and was characterized by a higher HIV prevalence among urban men. Previous studies have shown that the HIV epidemic in the general population of southern India is driven by sex work [9,10]. The sex work structure in the three districts underscores the heterogeneity of their HIV epidemic [11]. Data from integrated behavioural and biological assessments conducted in the three districts among female sex workers (FSWs) [11] have shown that FSWs are predominantly either brothel or home-based in Belgaum, while they are primarily home-based in Bellary, and street-based in Mysore. Belgaum FSWs have the highest median client volume, followed by Bellary and Mysore. Median age at sexual debut among FSW in Belgaum, Bellary and Mysore is 14, 15 and 16 years respectively, with a very high number of FSWs in sex work for more than 5 years in Belgaum and Bellary (65%) compared to Mysore (35%) [11]. The south-north gradient observed in the sex work structure is in line with the HIV prevalence in the general population in these three districts. The rural predominance of HIV in Belgaum may probably be due to predominantly rural sex work and the presence of traditional sex workers (Devadasis) based in rural areas [12]. The mobility of sex workers from Belgaum to large urban centers of Maharashtra for sex work is also likely to have brought HIV into the rural community in Belgaum [13]. The higher urban HIV prevalence in Bellary might be related to urban sex work and the presence of a large number of mining and chalking industries in urban Bellary, providing a large source of potential clients for FSW. This dynamic though needs further study.

The hypothesis that the epidemic in Karnataka progressed more rapidly in the northern districts of Karnataka compared to southern Karnataka may be further substantiated by the results of this study. There is however no confirmed reports on the date of HIV introduction into these districts and mathematical modelling analyses suggests that HIV was introduced in Mysore and Belgaum at around the same time in 1985 [14]. Nevertheless, there is some evidence supporting this hypothesis. Indeed, the sex work structure in northern Karnataka seems to favour more rapid spread of HIV compared to southern Karnataka [15]. In addition, mathematical modeling of the HIV epidemic in four cities in Africa has shown that with generalized epidemics where control programmes are in place, the prevalence of curable STIs either remains constant or reduces, and the prevalence of HSV-2 increase as the epidemic progresses, while the prevalence of curable STIs is higher during the early phase of the epidemic [16]. With the availability of effective control programmes in all these three districts, the higher prevalence of HIV and HSV-2 and lower prevalence of curable STIs in Belgaum may suggest a more generalized and mature epidemic. With a higher prevalence of curable STIs, Mysore seems to have a more concentrated HIV epidemic among the core group and an early phase epidemic. Information on the proportion of people with HIV who are eligible for antiretroviral drugs (i.e. with low CD4 counts) would be useful to further confirm the phase of the epidemic in these districts. In addition, with the presence of an effective control programme for curable STIs in Belgaum and Bellary, focusing on interventions against HSV-2 may have an important role in HIV prevention in these two districts, especially among the lower risk general population [16,17].

This study showed that Non-Muslim men had a higher HIV prevalence inspite of having a lower level of HIV risk behavior. A very low proportion of Non-Muslim men were circumcised. This strengthens the view that circumcision is independently associated with reduced risk of HIV compared to those not circumcised [18-21]. There was no significant difference of HIV risk behavior among Muslim and non-Muslim women. However, being a Muslim woman was protective for HIV. Given the nearly universal circumcision among Muslim men, it is very likely that Muslim women have circumcised male partners which may not be the case for non-Muslims, indirectly protecting Muslim women from HIV. However, information on circumcision among partners of women would help substantiate this hypothesis.

Among men the other risk factors for HIV included ever having paid for sex with a woman and ever having had a STI which were expected since they are classical risk factors for HIV. Among women, having had more than one sexual partner during their life time and having ever used a condom were significantly associated with HIV. The reverse association between condom use and HIV is consistent with the National Family Health Survey – 3 (NFHS-3) findings [22] and the Bagalkot survey [6]. This may be due to the fact that high risk women might have been infected with HIV and then started using condoms [8]. A similar general population study conducted in Guntur district in the neighbouring state of Andhra Pradesh showed a similar reverse association [21] among those who used condoms sometimes, often or always in the past six months substantiating the fact that they were infected with HIV before starting to use condoms, a phenomenon which the authors refer to as risk compensation. Being widowed/separated/deserted was the strongest predictor of HIV among women. These women tend to be socially and culturally isolated and are likely to be pressurised to meet the needs of their family. Such women might be forced into situations which make them vulnerable for sexual exploitation indirectly rendering them vulnerable for HIV. Even among FSWs, previous studies in India have shown a significant association between HIV and women with dissolved marriage [11,23].

This study has limitations. Because of social desirability bias, risky sexual behaviour may have been under-reported. There is evidence for social desirability bias in all the three districts where the underreporting of risky sexual behavior was significant in face-to-face interviews compared to polling booth surveys (PBS: anonymous group interviews) [14,24-27]. PBS conducted in parallel to the face-to-face interviews (FTFI) in our studies show a three to six-fold higher proportion of men reporting ever having paid for sex (Mysore: 1.8% in FTFI vs 6.2% in PBS, Belgaum: 2.1% in FTFI vs 12.9% in PBS and Bellary: 1.7% in FTFI vs 8.0 in PBS) [25-27]. However, this is unlikely to have affected the results of the current study, since the bias would likely be similar in all three districts, considering the uniform cultural and social attitudes towards risk behaviour in these communities. The overall response rate for biological samples in the three districts was 72.5% (range: 69.9% to 76.5%). This is relatively high compared to the general population survey conducted in Bagalkot (60.0%) [6], but lower than in the Guntur study (91.2%) [21]. The response rates were significantly different across gender and residence in urban vs. rural areas. The survey weights incorporated into the analysis took into account non-response for place of residence. Women were more likely to respond compared to men mostly because women were more often available at home than men during the survey. This difference is similar in all the three districts. Although the response rates were sub-optimal, it is unlikely to have any impact on the results of this study, at least for the sex-specific analyses.

The time difference for the conduct of the survey between Mysore and Bellary is around 1.5 years. This time difference is unlikely to have any effect on the results of this study since, even if HIV incidence could vary significantly over a short period of time, it is highly unlikely that HIV prevalence could vary a lot over a 1.5-year period, given the long duration of this infection.

In conclusion, this study highlights the importance of adopting district-specific approaches to HIV prevention, given the heterogeneous nature of the epidemic in the general population, coupled with the variations in the structure and organization of sex work, especially in a culturally and socially diverse country like India. Understanding this heterogeneity is important to develop and implement effective interventions. Identification of the phase and maturity of local HIV epidemics would help to adapt interventions to local situations.

## Competing interests

The authors declare that they have no competing interests.

## Authors' contributions

PB was responsible for data analysis and writing the manuscript and coordinating the data collection in Mysore. SPR contributed to data analysis and interpretation of results. SBM coordinated data collection in Belgaum and Bellary. BMR, RGW and CML contributed to the study design, development of questionnaires, overall supervision of the study and the interpretation of data. SM and JFB participated in the design of the study and contributed to the interpretation of the results. MA was responsible for the study design, development of questionnaires, data collection, interpretation of the data and supervision of the study. All of the authors participated in revising the manuscript critically for intellectual content and approved the final version of the manuscript.
